# Editorial: Digital media use in early childhood—contextual factors, developmental outcomes, and pathways

**DOI:** 10.3389/frcha.2025.1627511

**Published:** 2025-06-13

**Authors:** Margarete Bolten, Eva Unternaehrer

**Affiliations:** ^1^ Child and Adolescent Psychiatric Clinic, Luzerner Psychiatrie AG, Lucerne, Switzerland; ^2^ Department of Clinical Research, Faculty of Medicine, University of Basel, Basel, Switzerland; ^3^ University Psychiatric Clinics Basel (UPK), University of Basel, Basel, Switzerland

**Keywords:** early childhood, screen time (ST), technoference, parenting (MeSH), digital media, infants

**Editorial on the Research Topic**
Digital media use in early childhood—contextual factors, developmental outcomes and pathways

## Introduction

The digitalization of our world and the associated changes in daily life are influencing how infants and toddlers grow up. Digital media, the internet, and screens of various sizes have become an integral part of most families' lives, often with multiple devices per household. Especially the rapidly emerging portable devices, such as smartphones, tablets, or laptops, provide unrestricted access to screen media anywhere and anytime. It is therefore hardly surprising that the daily use of digital screen devices has not only increased in the general population in the last decade, but also in very young children (1). For instance, screen time in American families has risen in infants (0–2 years) from 1.32 h per day in 1997 to 3.05 h per day in 2014, which included an average of 2.62 h of television viewing and 0.37 h on mobile devices. According to the most recent Common Sense Census, children under the age of 2 spend approximately 1 h per day on a screen (2). Surveys on parental use of screen technologies for their children show that care-takers often use screen media to distract their children from emotional or physical discomfort—to calm them down, to stop tantrums, to enable feeding, or to put them to sleep (3). Other reasons for media use are: keeping children occupied when parents are doing housework, trying to work, are out at an event, or simply if they need a break (4).

Early childhood is a sensitive developmental period during which close, responsive relationships and rich multisensory experiences are essential for healthy cognitive, socio-emotional, language and motor development. Three particularly salient risks have emerged in this context: (a) the direct use of screen media by young children and (b) technoference—the disruption of parent-child interactions due to parental media use, and (c) the lower use of embodied motor activities. The developmental implications of these patterns remain insufficiently understood and warrant further investigation.

There has been concern that direct screen media exposure may harm children's development, especially if screen use starts very early in life and takes up as lot of time in the child's life. The first 3 years of a child's life are a period of rapid brain development, including cognitive, language, social, and motor processes (5). Within 36 months, the child turns from a completely dependent newborn to a remarkably complex individual who shares thoughts, feelings, and intentions with others, expresses him- or herself by using words, and understands social signals and norms. The World Health Organization (WHO), and many other organizations invested in early childhood health and development discourage screen media use for children younger than 18 or 24 months, respectively, and recommend limiting sedentary screen media exposure for children between 2 and 5 years to 1 h per day restricted to high-quality screen content (6, 7). However, recent cohort studies indicate that many families do not follow these guidelines (8). In the study of Putnick et al. (9) about 17% of the assessed infants started using screen media during the first year of life with approximately 1 h per day, increasing to 2–3 h per day at the age of 2–3 years. The increased use of digital media in early childhood is associated with a displacement of time spent in physically active or social play, which is essential for sensorimotor, cognitive, and socio-emotional development (10, 11). Motor activities in infancy and early childhood contribute not only to gross and fine motor skills but also to the development of executive functions, self-regulation, and spatial cognition (12). Sedentary screen use may thus reduce opportunities for children to explore their environment, engage in goal-directed movement, and interact with caregivers or peers through physically co-regulated play. These limitations can, in turn, have downstream effects on developmental cascades, particularly in vulnerable populations. The indirect effects of screen exposure include also parental technoference, which describes parental use of technology during a social interaction with their child. Parents act as role models for mobile screen media use and show reduced responsivity and availability for their children when using a smartphone, with potential negative consequences for child mental wellbeing (13).

With this Research Topic, we aimed to deepen the scientific discourse on how digital media use may affect early development, while considering the complex and dynamic systems within which children grow. The contributions span a variety of methodologies—including longitudinal designs, process evaluations, parental surveys, behavioral observations, and population-based cohort studies—and offer a multifaceted view of the topic.

Looking at potential mechanisms and contextual factors in the association between screen use and child development, Konok et al., investigated how parental use of digital media for emotional regulation is associated with the development of self-regulatory skills in children. Their study highlights a potentially important intergenerational pathway by which media use patterns are transmitted and internalized.

Other contextual factors that were featured prominently were family routines and parental strategies. Drawing on data from a large Canadian cohort, the study by Lien et al. explored the role of limit-setting and routines in shaping children's screen use during the pandemic. Meanwhile, Fitzpatrick et al. demonstrated how parents' own media habits may influence children's global developmental outcomes. Finally, the research group of Paulus et al. provided further evidence for the role of the familial context in shaping digital media use among children aged 0–4 years. Findings of this paper underscore that media habits do not arise in a vacuum but are embedded in a broader socio-emotional and relational framework that includes parental stress, parenting styles, and environmental factors.

Turning to the microsystem of parent-child interaction, Liszkai-Peres et al. and Chamam et al. examined the effects of mobile touchscreen device use on dyadic communication. While Liszkai-Peres et al. focused on how device use during interactions may degrade interaction quality, the comparative study on digital vs. non-digital distraction published by Chamam et al. provided nuanced insights into the varying impact of different types of parental inattention.

Finally, some studies also examined potential intervention approaches. In a process evaluation of a large-scale intervention, Schemmer et al. applied the RE-AIM framework, a widely used tool to evaluate the impact and sustainability of health interventions in real-world settings. RE-AIM stands for: Reach, Effectiveness, Adoption, Implementation, and Maintenance (14). Schemmer et al. assessed the implementation and effectiveness of a Germany-wide program aimed at preventing dysregulated screen time use in children under 3. This work emphasizes the importance of structural prevention efforts and implementation science in shaping healthy digital habits from the start. Complementing this, Fitzpatrick et al. identified effective parenting strategies for meeting screen time recommendations.

**Figure 1:**
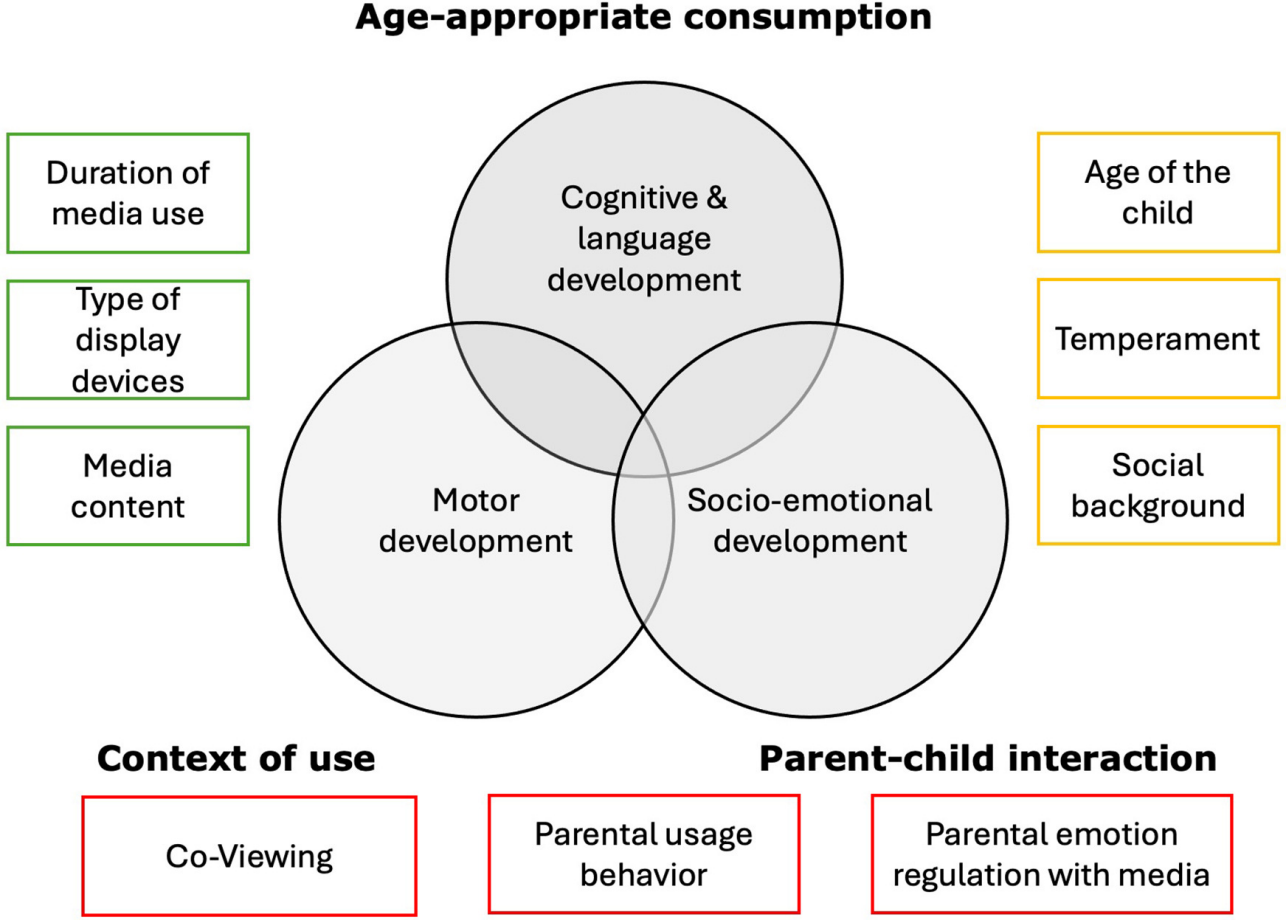
Overview of research findings on early childhood screen use: developmental outcomes and contextual factors.

Taken together, the articles in this Research Topic illustrate that the developmental effects of digital media in early childhood strongly depend on context of screen use. They highlight the need for integrative research approaches that consider parental characteristics, interaction quality, family systems, and longitudinal trajectories. Figure 1 illustrates the multifactorial influences of early screen use on infant development, focusing on three core developmental domains: cognitive & language development, motor development, and socio-emotional development. It provides a conceptual overview of how various contextual, individual, and environmental factors interact with digital media use in early childhood. The illustration is based on the assumption that developmental effects of screen use depend not only on how much but also on how, what, and with whom media is consumed—emphasizing the need for age-appropriate, co-regulated, and developmentally sensitive media use.

Far from demonizing digital media, these contributions call for a more differentiated understanding of its place in modern childhood—and of the protective and risk factors that modulate its effects.

We are grateful to the authors and reviewers who contributed their expertise to this Research Topic. We hope this issue stimulates ongoing dialogue, policy reflection, and research innovation aimed at promoting healthy development in a digital world.
